# Appraisals of stressors and common mental disorder from early to mid-adulthood in the 1946 British birth cohort

**DOI:** 10.1016/j.jad.2009.03.021

**Published:** 2009-12

**Authors:** Stephani L. Hatch, Gita Mishra, Matthew Hotopf, Peter B. Jones, Diana Kuh

**Affiliations:** a Department of Psychological Medicine, Section of General Hospital Psychiatry, Institute of Psychiatry, King's College London, UK; b Medical Research Council Unit for Lifelong Health and Ageing and the Medical Research Council National Survey of Health and Development, UK; c Department of Psychiatry, University of Cambridge and Cambridgeshire and Peterborough Mental Health National Health Service Trust, Cambridge, UK

**Keywords:** Depression, Anxiety, Stressful life events, Birth cohort

## Abstract

**Background:**

We examined the extent to which perceived life change following experiences of stressful life events, differentiated by type of stressor, influenced mental health during adulthood.

**Methods:**

The analytic sample of 2073 cohort members was drawn from the MRC National Survey of Health and Development, a sample followed since their birth in March 1946. Logistic regression was used to assess the relationship between stressors reported at 36 and 43 years and common mental disorder at 36, 43, and 53 years. Common mental disorder was measured using the Present State Exam at 36 years, the Psychiatric Symptom Frequency at 43 years, and the 28-item General Health Questionnaire at 53 years.

**Results:**

Data spanning across nearly 20 years suggest that stressors perceived to have contributed to a notable life change increased the likelihood of scoring above the cut off score for common mental disorder in comparison to stressors experienced without subsequent life change. Models were adjusted for gender, educational attainment, social class, relationship status, and past episodes of common mental disorder. This relationship appears to be most evident for proximal family and economic stressors and distal interpersonal relationship stressors experienced by close friends and relatives.

**Limitations:**

All study information is based on self-reports and details about the nature of the life change or cognitive attribution style were not available.

**Conclusions:**

Appraisals of changes following stressful life events may be more important than the occurrence of stressors alone in assessing the impact of stressful life events on adult mental health.

## 1 Introduction

While the significance of stress for common mental disorder has a well documented history across disciplines (Aneshensel, 1992; Brown and Harris, 1978; Turner and Lloyd, 2004; Dohrenwend and Dohrenwend, 1974; Pearlin et al., 1981), one rarely captured dimension of stress is the perceived change in daily life following the experience of a life event or chronic strain (i.e., perceived magnitude) (Dohrenwend, 2000, 2006). In addition, few studies have been able to show the long term impact of stressors on mental health throughout adulthood. In this paper we used prospective data spanning nearly 20 years from the Medical Research Council (MRC) National Survey of Health and Development (NSHD), a nationally representative population of men and women followed from their birth in England, Scotland, and Wales in March 1946 until age 53 years, to assess the impact of stressors with and without subsequent life change on mental health at multiple time points in early and mid-adulthood.

### 1.1 Stress process and life course perspectives

Stress process theory emphasizes that exposure to stressors includes both discrete life events (e.g., death of a relative or friend) and chronic strains (e.g., financial strain); that the stressors are not randomly distributed throughout the population; and that they are deeply rooted in the structural contexts of people's lives (Kessler, 1979; Pearlin, 1989; Aneshensel, 1992). Situated within a sociological framework for mental health, stress process theory focuses on understanding the social structural origins of common mental disorder (Pearlin, 1989; Aneshensel and Phelan, 1999). Because social status positions are hierarchically organised, they impact health by influencing access to resources, opportunities, achieved statuses, and power (Link and Phelan, 2000; Kuh et al., 2004). One process by which stressors come to impact health is through experiences that arise in relationship to ascribed statuses (e.g., gender, race/ethnicity) and achieved statuses (e.g., own social class) (Hatch, 2005; Lantz et al., 2005; Pearlin et al., 2005). This is probably most evident through stressors that are linked to ascribed social statuses because they are a constant, rather than modifiable, and linked to highly salient social roles and identities (Pearlin et al., 2005).

### 1.2 Disaggregating stressors by type

The use of cumulative stressor scores assumes that different types of events have characteristics in common that determine the nature and extent of their impact. In contrast, disaggregating stressors by type takes into consideration the context of major social domains and the potential for differential impact of stressors on health (Wheaton, 1999). For example, stressors experienced within family and work domains may be tied to more salient and more valued social roles than stressors representing more peripheral social roles (Pearlin et al., 2005; Thoits, 1999). Findings from two studies of women in the NSHD cohort found increased reporting of work and family-related life stressors experienced in mid-adulthood was associated with increased common mental disorder (Hardy and Kuh, 2002; Kuh et al., 2002).

### 1.3 Perceived impact of adversity

Dohrenwend (2000, 2006) suggests that the characteristics of commonly experienced stressors, such as their impact on change in usual or daily activities are essential to understanding the social effects of stress on mental health. Negative life changes following a stressful experience may signify the process through which stressors can impede the achievement of valued goals that are central to social role coherence and purpose (Aneshensel, 1992; Horwitz, 2007). Thus, the central hypothesis is that the greater the negative changes in situations following a negative event, the greater the likelihood that common mental disorder will develop (Dohrenwend, 2000).

Stemming from the work of Brown and colleagues (i.e., Life Events and Difficulties Schedule), methodological developments over the years have focused on objectively rating the stressfulness of an event in an effort to avoid recall bias due to “effort after meaning” (coined by Bartlett, 1932) among psychiatric cases attempting to explain their illness (Brown and Birley, 1968). Various studies that undertook these or similar methods showed that commonly experienced stressors rated for having substantial magnitude, referencing subsequent changes in daily life, were more strongly related to psychiatric disorder (Brown and Harris, 1978; Shrout et al., 1989; Brown, 1998; Stueve et al., 1998) than stressors with no assessments of consequent life change. This in-depth approach was feasible because they were conducted on small community or treatment samples. In contrast, large surveys tend to rely on simple checklists of potentially stressful life events, with few capturing subsequent life change, because they are more economical and practical for studies on the general population.

### 1.4 Study aims

The first aim of this study is to examine the effect of the accumulation of stressors on common mental disorders. The second aim of this study is to test Dohrenwend's (2000) hypothesis that stressors reported to have led to life changes have a stronger association with common mental disorder than those stressors reported not to have led to life changes. We posit that this will be evident in the relationships between accumulated stressors reported to have occurred in the year prior to the interview and current common mental disorder, as well as the long term impact of stressors on mental health across adulthood. A third aim is to determine whether or not any long term impact of stressors on mental health can be differentiated by the type of stressors or modified by social statuses, such as gender or social class.

## 2 Methods

### 2.1 Study participants

Participants were drawn from the MRC NSHD , a birth cohort study stratified by social class and initially consisting of 5362 people selected from all births that occurred in England, Scotland, and Wales during one week in March 1946 (Wadsworth, 1991). At the last home visit in 1999, at age 53 years, information was collected from 3035 individuals (83% of the target sample and 57% of the original cohort). The remaining cohort members had either died (9%), emigrated or living abroad (11%), previously refused to participate in the study (12%) or were untraced (6%) (Wadsworth et al., 2006). At this time the cohort was shown still to be a representative sample, in most respects, of the UK population born singly and within marriage in the immediate post-war era (Wadsworth et al., 2003). There was a disproportionate loss to follow-up in adulthood among the most disadvantaged (e.g., lower educational level and social class). Information used in this study was collected by research nurses when study members were 36, 43, and 53 years.

### 2.2 Measures

#### 2.2.1 Measures of common mental disorder in adolescence and adulthood

For ease of interpretation, common mental disorder was captured in this analysis through identifying groups that met the criteria for ‘caseness’ (scored above the respective cut off value) and this is implied in references to being a case on the various adult measures.

*Adolescent common mental disorder* refers to teachers' observations of students' anxious and depressive symptoms collected at ages 13 and 15 years. In the first item, students were characterised as ‘not at all anxious, apprehensive or fearful,’ ‘somewhat anxious, apprehensive, or fearful,’ or ‘very anxious, apprehensive or fearful’. In the second item they were described as ‘usually happy and contented,’ ‘generally cheerful and in good humour, or ‘usually sad or gloomy’. Items were recoded into never being assessed as either anxious or sad at either 13 or 15 years versus being assessed as very or somewhat anxious or usually sad at either or both time points to provide a conservative estimate of mental health in adolescence. The use of these socially oriented assessments of mental health, in comparison to personality indicators, has been previously established research on adolescent affect in the NSHD (see Van Os and Jones, 1999; Paykel et al., 2001; Colman et al., 2007, 2009; Hatch and Wadsworth, 2008).

*Common mental disorder in adulthood* was measured with the Present State Examination (PSE) at 36 years, the Psychiatric Symptom Frequency (PSF) scale at 43 years, and the 28-item General Health Questionnaire (GHQ-28) at 53 years. A shortened version of the PSE was administered by trained nurses at 36 years to obtain standardised interview ratings of low mood, anxiety, and phobia symptoms in reference to one month prior to the interview. A computer-generated, previously validated categorical variable (Wing et al., 1974) was created from this 48-item diagnostic assessment through an index of definition (ID) where 4 or higher (more inclusive of sub-threshold cases in the general population) was taken as evidence of common mental disorder (see Rodgers and Mann (1986) for further details on the criterion-related validity of the PSE in this sample) (11.9% of the population).

Trained research nurses captured mental health at age 43 years by administering the PSF scale containing a 19-item scale measuring current and recent (past 12 months) depression and anxiety symptoms (Rodgers, 1996). A cut-off score of 22 (range, 0 to 95; 13.4% of the population), previously determined to define high risk of clinical disorder at 43 years, was used for a conservative estimate of common mental disorder based on past research on the criterion-related validity of the PSF (Lindelow et al., 1997).

The common mental disorder outcome at 53 years was assessed by the GHQ-28 that focuses on symptoms of anxiety and depression (Goldberg and Hillier 1979). The GHQ-28 is a self-administered screening questionnaire for detecting recent (past few weeks) symptoms of common mental disorder. Each individual item was scored using a 1 to 4 point Likert scale and recoded into binary values. The cut-off score used for this study was determined by the majority of validity studies of the GHQ-28 suggesting a threshold of 6 for being a case (16.8% of the population) (Goldberg and Williams, 1988).

*Past common mental disorder* has been shown to be important aspect of understanding the relationship between accumulated stressors and common mental disorder (see Monroe and Harkness, 2005 for a review). Each measure of common mental disorder was entered as a continuous indicator. The PSF at 43 years was subjected to a log transformation to improve distribution.

#### 2.2.2 Measures of stressors

Each stressor was captured in checklists at 36 and 43 years and referred to experiences in the year prior to the interview. Descriptive information for each item at 36 years and 43 years is presented in Table 1. Stressors were categorised into two types: (1) family and economic/work stressors to self, children, or spouse and (2) interpersonal relationships stressors indicated by stressors experienced by close friends and relatives.

Following questions about the occurrence of the checklist items, respondents were asked to indicate whether or not each stressor led to a life change, and this information was used to create three categories of accumulated stressors: (1) no stressors, (2) stressors with no life change, and (3) stressors with life change.

#### 2.2.3 Potential confounders

*Social class* was a categorisation of the cohort member's or partner's most recent occupational classification five categories (professional, managerial and intermediate, skilled manual, semi-skilled manual and unskilled) according to the Registrar General and captured at four time points from age 26 to 53 years. The six categories were recoded into a dichotomised variable indicating non-manual versus manual to capture the general social class position occupied throughout adulthood and improve distribution. Based on findings from past studies that showed differential experiences of stressors by gender and social class (Turner et al., 1995; Turner and Avison, 2003), these factors were considered separately as potential modifiers or confounders of the effect of stress on mental health in adjusted models.

Other potential confounders included educational attainment and relationship status. *Educational attainment by 2*6 year*s* was categorised as no qualifications attempted, vocational qualifications was classified according to the Burnham scale (Department of Education and Science, 1972) and grouped into the following: no qualification, below ordinary secondary qualifications (e.g., vocational qualifications), ordinary level qualifications (‘O’ levels or their training equivalents), advanced level qualifications (‘A’ levels or their equivalents ) or higher education. *Relationship status* distinguished respondents who were married from those who were separated, divorced, or widowed at each time point.

#### 2.2.4 Analytic strategy

The sample available to assess whether or not there was a relationship between stress accumulation and stress appraisal (i.e., reported with or without subsequent life change) had a differential impact on mental health in adulthood totalled 2073. Logistic regression was used to calculate the odds of meeting the criteria for common mental disorder as measured by the PSE at 36 years, the PSF at 43 years, and the GHQ-28 at 53 years. To address the first aim, we examined the association between the number of reported stressors at 36 and 43 years from the past year and concurrent and prospective assessments of common mental disorder. Addressing the second aim involved comparing the effect of the appraisal of stressors at 36 and 43 years from the past year, reported with and without life change, on both concurrent and prospective assessments of common mental disorder. Stressors reported without life change was used as the reference category in all models, as opposed to the no stressors category, in order to make direct comparisons of stressors reported with and without life change. In the first set of models (Model 1), unadjusted associations were tested between the type of stressor at each time point and the three outcomes. The second set of models (Model 2) assessed the effect of both types of stressors reported at 36 years on each of the three outcomes and were partially adjusted for gender, educational attainment, social class, and relationship status. The third set of models (Model 3) tested the effect of both types of stressors reported at 43 years on the outcomes at 43 and 53 years. In Model 3, past common mental disorder was added to test the fully adjusted models. To address the third and final aim, stressors reported at 36 and 43 years were entered together in a model with caseness on the GHQ-28 at 53 years as the outcome to assess whether or not any long term impact of stressors on mental health can be differentiated by the type of stressors, family and economic and interpersonal relationship, or modified by social statuses, such as gender or social class. Further, interaction terms between stressors and gender and social class were entered into these models to test possible differences in the effect of stressors on mental health by these factors.

## 3 Results

### 3.1 Descriptive statistics

Descriptive characteristics of study variables are presented in Table 2. Because different items were used to assess common mental disorder at each age, comparisons across measures were not possible.

### 3.2 Accumulation of stressors at 36 years and common mental disorder at 36, 43, and 53 years

Table 3 shows the unadjusted and adjusted odds ratios for scoring above the cut off score on common mental disorder outcomes throughout adulthood by number of past year stressors reported at 36 years. All categories representing the number of stressors reported at 36 years were associated with being a case on the PSE at 36 years in comparison to no stressors. The associations were not attenuated in the partially adjusted model (Model 2) or the fully adjusted model (Model 3).

There was no association between reporting one or two stressors at 36 years in comparison to reporting no stressors and being a case on the PSF at 43 years or being a case on the GHQ-28 at 53 years in the unadjusted or adjusted models. In contrast, reporting either three stressors or four or more stressors at 36 years increased the odds of being a case on the PSF at 43 years in the unadjusted model (Model 1) and partially adjusted model (Model 2). Only reporting four or more stressors at 36 years in comparison to reporting no stressors was associated with being a case on the GHQ-28 at 53 years. In fully adjusted models (Model 3), reporting four or more stressors at 36 years was associated with an increase risk of being a case on the PSF at 43 years and being a case on the GHQ-28 at 53 years.

### 3.3 Accumulation of stressors at 43 years and common mental disorder at 43 and 53 years

Reporting two, three, and four or more stressors at 43 years in comparison to reporting no stressors was associated with an increase in the odds of being a case on the PSF at 43 years and being a case on the GHQ-28 at 53 years in the unadjusted and partially adjusted models (Model 1 and Model 2). For being a case on the PSF at 43 years, these associations were weakened, but not attenuated by adjusting for past common mental disorder in the fully adjusted model (Model 3). In contrast, there was no association between these categories of accumulating stressors reported at 43 years and being a case on the GHQ-28 at 53 years in Model 3.

### 3.4 Differentiating stressors at 36 years by type and common mental disorder at 36, 43, and 53 years

Table 4 shows the unadjusted and adjusted odds ratios for scoring above the cut off score on mental health outcomes throughout adulthood by past year stressors by type reported at 36 years. In separate unadjusted models (Model 1), family and economic stressors reported with life change (odds ratio (OR) = 2.05, 1.33–3.16) and interpersonal relationship stressors reported with life change (OR = 1.64, 1.16–2.31) resulted in greater risk of being a case on the PSE at 36 years in comparison with the reference category of reporting these stressors without subsequent life change. Model 2 included adjustments for gender, educational attainment, occupational social class, and relationship status. In comparison to reporting stressors with no subsequent life change stressors of family and economic stressors (OR = 1.97, 1.26–3.08) and interpersonal relationship stressors reported with life change (OR = 1.60, 1.11–2.24) were associated with being a case on the PSE at 36 years. Further adjustment for past common mental disorder in Model 3 failed to attenuate this association.

Similarly, family and economic stressors reported with life change (OR = 1.68, 1.07–2.63) and interpersonal relationship stressors reported with life change (OR = 1.48, 1.06–2.08) at 36 years were associated with being a case on the PSF at 43 years in comparison to reporting stressors with no life change (Model 1). Only interpersonal relationship stressors reported with life change (OR = 1.42, 1.00–2.01) resulted in a greater risk of being a case on the PSF at 43 years in comparison to reporting no life change after partial adjustments in Model 2. However, after adjustment for past common mental disorder in Model 3, there was no association between interpersonal relationship stressors reported with life change and the PSF at 43 years.

There was no association found between family and economic stressors and caseness on the GHQ-28 at 53 years in Model 1. In contrast, interpersonal relationship stressors reported with life change (OR = 1.68, 1.24–2.28) were associated with being a case on the GHQ-28 at 53 years. The partially adjusted model (Model 2) and the fully adjusted model (Model 3) continued to show an association between interpersonal relationship stressors reported with life change and being a case on the GHQ-28 at 53 years in comparison to stressors reported with no life change (OR = 1.65, 1.21–2.25 and OR = 1.42, 1.02–1.97, respectively).

### 3.5 Differentiating stressors at 43 years and common mental disorder at 43 and 53 years

Unadjusted and adjusted models for the impact of stressors captured at 43 years on the PSF at 43 years and the GHQ-28 at 53 years also were shown in Table 4. In unadjusted models (Model 1), reporting family and economic stressors with life change (OR = 1.51, 1.07–2.21) and interpersonal relationship stressors with life change (OR = 2.12, 1.53–2.93) was associated with being a case on the PSF at 43 years in comparison to reporting stressors with no life change. Family and economic stressors reported with life change at 43 years (OR = 1.43, 1.01–2.03) and interpersonal relationship stressors at 43 reported with life change (OR = 2.03, 1.46–2.84) were associated with being a case on the PSF at 43 years in Model 2. Only the association between interpersonal relationship stressors and being a case on the PSF at 43 years (OR = 1.93, 1.37–2.71) persisted in the fully adjusted model (Model 3).

There was no unadjusted association between family and economic stressors with life change at 43 years and being a case on the GHQ-28 at 53 years. In contrast, there was an association between interpersonal relationship stressors reported with life change at 43 years and being a case on the GHQ-28 at 53 years in comparison to reporting no change (Model 1, OR = 1.57, 1.15–2.14). This relationship was slightly attenuated in Model 2 (OR = 1.45, 1.05–1.98), but there was no association after adjusting for past common mental disorder in Model 3.

### 3.6 Assessing the long term and independent effect of stressors by type on the GHQ-28

Table 5 shows the adjusted odds ratios for being a case on the GHQ-28 at 53 years by stressor type and life change following the stressor reported at two time points, 36 and 43 years. In Model 1, interpersonal relationship stressors with life change at 36 years and at 43 years had an independent effect on the increased likelihood of being a GHQ-28 case at 53 years in comparison to those who the stressors with reported no life change (OR = 1.59, 1.16–2.17 and OR = 1.41, 1.03–1.93, respectively). There was no association between family and economic stressors at 36 years or at 43 years reported with life change and being a case on the GHQ-28 at 53 years. In Model 2, mutual adjustment for stressors at 36 and 43 years and additional adjustment for past common mental disorder attenuated the association between the interpersonal relationship stressors at 43 years. By comparison, interpersonal relationship stressors at 36 years reported with life change were associated with increase likelihood of being a GHQ-28 case at 53 years in comparison to stressors reported with no life change (OR = 1.41, 1.02–1.96).

Tests for interactions between gender or social class and stressors at either 36 years or 43 years on common mental disorder were not significant at the 10% level for any of the outcomes.

## 4 Discussion

This analysis considered the extent to which stressors differentiated by perception of subsequent life change and by type of stressor was associated with common mental disorder captured concurrently and prospectively throughout adulthood. This study found differential effects of stress on the likelihood of common mental disorder from early to mid-adulthood by the number of stressors accumulated, by type, and whether or not the stressor was reported with subsequent life change. As expected, there was evidence that stressors reported with life change resulted in an increase risk of common mental disorder in comparison to those reported without life change in which models adjusted for gender, educational attainment, occupational social class, relationship status, and past common mental disorder. This relationship appears to be most evident in the more proximal experience of family and economic stressors and the more distal experience of interpersonal relationship stressors.

As shown in these prospective data spanning nearly twenty years, stressors occurring to close friends and relatives appear to have a longer term impact on mental health than those experienced by more immediate family (husband and children) and to self. The results from the final model suggest that the conflicts and changes in health of close friends and relatives reported at 36 years represent hardships that failed to resolve from early to mid-adulthood. As noted in Horwitz (2007), it is likely that the lasting impact of these stressors is due to impediment of the valued goals and/or social roles of the individual. Any short term impact of family and economic stressors on mental health appears to dissipate over time. In contrast, interpersonal relationship stressors perceived to have contributed to a notable change in daily life appears to have had a long term deleterious effect on the likelihood of common mental disorder in mid-adulthood.

The long term influences of interpersonal relationship stressors on mental health may be an indication of a predisposition to or recently remitted common mental disorders that may be coupled with poor relationship skills and/or close friends and relatives (i.e., a stress generation effect) (Hammen, 1991). The perceptions of subsequent life change may reflect a negative cognitive style that contributes to an increase in the likelihood of experiencing stressful life events (Safford et al., 2007). It also may be that the reported stressors represented a single hardship that could be characterized by its persistent and continuous effects over time (Hatch, 2005) or these stressors may be a part of a chain of contingencies in which these types of hardships are the beginning of a series of unfolding hardships (i.e., stress proliferation) that are different but related to the initial stressor (Pearlin, 1989).

Finally, unexpectedly and somewhat contrary to past evidence (see Hatch and Dohrenwend, 2007 for a review), there was no differential distribution or effect of stress on mental health by gender or social class in this sample. While our finding regarding gender is consistent with Costello et al. (2002) and Turner et al. (1995), who found no gender difference in the reporting of stressors, the majority of the evidence suggests that women tend to report more stressors, particularly those related to significant others (Bieliauskas et al., 1995; Kessler and McLeod, 1984; Turner et al., 1995). Our findings that showed no differences in the distribution of stressors by social class were also inconsistent with several studies that have found that individuals occupying disadvantaged socioeconomic positions report more experience of stressors (MacLeod and Kessler, 1990; Turner et al., 1995).

### 4.1 Study considerations and limitations

One important consideration in stress research is whether or not the individual's mental health state at the time of the interview resulted in differential reporting of life change following stressors. Among the cases of common mental disorder, there was no evidence of elevated stressors being reported with life change than without life change at either 36 years or 43 years. Because no more detail about the nature of the perceived life change was available and the information was collected through self-report and not corroborated by external raters, the estimates should be interpreted with some caution. However, the pattern of results showing a greater effect size for stressors reported with life change than without across different measures of common mental disorder suggests this simple measure is informative in understanding the relationship between stress and mental health. This is consistent with past studies that found stronger associations between subjective stressors and behavioural outcomes and psychological adjustment (Darr and Johns, 2008; Froggio and Agnew 2007) in comparison to more objectively measured stressors.

There are limitations that are relevant to studies capturing stressors and their relationship to mental health; some that deserve a mention in the context of this analysis, while others are discussed in detail elsewhere (see Dohrenwend, 2006; Hatch and Dohrenwend, 2007). Because the majority of the evidence linking adversity to health outcomes relies on the accuracy of retrospective reporting and temporal ordering of the occurrence of stressors, reliability in reporting and recall bias are core issues and vary by stressor (Turner and Lloyd, 2004). In this study, recollection periods of one year or less reduce recall biases and these prospective data allow for better identification of temporal order. In addition, we recognise that the intra-item variability, combining several potentially distinct stressors (e.g., accident, illness and injury) into in a single checklist item constrains the assessments of individual stressors beyond the descriptive level and potentially undermines the construct validity of the individual items (Dohrenwend, 2006). Thus, it was difficult to assess the effects of individual stressors on mental health for this analysis. Another limitation of this study is the inability to test the stress process model, namely the potential mediating factors, such as social support and mastery (i.e., sense of control). Finally, although the measures of common mental disorder generally captured symptoms of depression and anxiety, we did not have repeated measures in these data. In addition, controlling for past and concurrent mental health state, this analysis does not rule out reverse causation or determine whether the measures signify a psychological state or trait.

### 4.2 Conclusion

Despite these limitations, this analysis suggests that the effects of stressors on mental health appear to vary by type and perceived life change. While these data do not provide extensive detail about the nature of the reported change suggested by Dohrenwend (2000, 2006), differences in the effect of stressors with life change, versus no life change, on mental health suggests support for the position that the perceived impact of stressors are important for understanding deleterious mental health outcomes.

This prospective study showed that life change following stressful experiences matters for mental health and appears to be more important for stressors stemming from interpersonal relationships in comparison to family and economic events and chronic strains. Furthering our understanding of ways in which stress consistently comes to impact health necessitates more detailed assessments of the nature of stressors, the timing of the experience, perceived changes in daily life as stressors accumulate, and how the impact of stress on health may change over the life course.

## Role of the Funding Source

The National Survey for Health and Development is funded by the Medical Research Council (MRC). GM and DK are supported by the MRC. The MRC had no further role in study design; in the collection, analysis, and interpretation of data; in the writing of the report; and in the decision to submit the paper for publication.

## Conflict of Interest

None.

## Figures and Tables

**Table 1:** Description of stressors by type administered by checklist at 36 years and 43 years (*N* = 2073).

	At 36 years	At 43 years
	*N* (%)	*N* (%)
*Family and economic stressors*		
Victim of robbery or assault	125 (6.0)	55 (2.7)
Family crisis	300 (14.5)	–
Crisis or serious disappointment at work	276 (13.3)	150 (7.2)
Crisis or serious disappointment at work for spouse	–	114 (6.3)
Serious illness developed or diagnosed	–	57 (2.7)
Accident resulting in serious injury for month or more	–	113 (5.5)
Serious accident, injury, or assault to spouse	–	126 (6.9)
Serious health or behaviours problem with child	–	338 (18.7)
Serious disagreement or betrayal by spouse	–	133 (7.3)
Employment loss	–	152 (7.8)
Spouse's employment loss	–	142 (7.8)

*Interpersonal relationship stressors*		
Close friend or relative in serious accident or received a serious injury	236 (11.4)	–
Close friend or relative hospitalised or serious illness	930 (44.9)	544 (26.2)
Death of a close friend or relative	851 (41.1)	722 (34.8)
Close friend or relative separated or divorced	552 (26.6)	–
Serious disagreement or betrayal by close friend or relative	–	186 (9.0)
Loss of contact with close friend or relative	–	146 (7.0)

**Table 2:** Description of all study variables (*N* = 2073).

	*N*	%
Gender		
Female	1022	49.3
Male	1051	50.7
Educational attainment by 26 years		
No qualifications	748	36.1
Vocational qualifications	156	7.5
Ordinary level qualifications	440	21.2
Advanced level qualifications or above	730	35.2
Social class		
Manual	704	34.0
Non-Manual	1369	66.0
		
*Relationship status*		
Married		
At 36 years	1774	85.6
At 43 years	1716	82.8
At 53 years	1659	80.0
Separated or divorced or widowed		
At 36 years	35	1.7
At 43 years	246	11.9
At 53 years	316	15.2
Total number stressors at 36 years		
No stressors	455	21.9
One stressor	585	28.2
Two stressors	591	28.5
Three stressors	301	14.5
Four or more stressors	141	6.8
Total number stressors at 43 years		
No stressors	547	26.4
One stressor	669	32.3
Two stressors	467	22.5
Three stressors	255	12.3
Four or more stressors	135	6.5
Family and economic stressors at 36 years		
None	1478	71.3
One or more stressor, no life change	316	15.2
One or more stressor, with life change	279	13.5
Interpersonal relationship stressors at 36 years		
None	582	28.1
One or more stressor, no life change	1187	57.3
One or more stressor, with life change	304	14.7
Family and economic stressors at 43 years		
None	1217	58.7
One or more stressor, no life change	531	25.6
One or more stressor, with life change	325	15.7
Interpersonal relationship stressors at 43 years		
None	853	41.1
One or more stressor, no life change	902	43.5
One or more stressor, with life change	318	15.3
		
*Common mental disorder*		
Adolescence (13 and 15 years)		
Case	150	7.2
Non-case	1924	92.8
PSE at 36 years (cut off score ≥ 4)		
Case	247	11.9
Non-case	1826	88.1
PSF at 43 years (cut off score ≥ 22)		
Case	277	13.4
Non-case	1796	86.6
GHQ-28 at 53 years (cut off score ≥ 6)		
Case	348	16.8
Non-case	1725	83.2

Note: As different items were used to indicate common mental disorder at the different ages, percentages from these measures are not comparable.

**Table 3:** Unadjusted and adjusted odds ratios (OR, 95% C.I.) for scoring above the cut off score on common mental disorder outcomes by number of reported stressors at 36 or 43 years (*N* = 2073).

		PSE at 36 years		PSF at 43 years		GHQ-28 at 53 years
	OR	(C.I.)	OR	(C.I.)	OR	(C.I.)
*Stressors at 36 years*						
Model 1 (unadjusted)						
No stressors (ref.)	1		1		1	
One stressor	1.71	(1.08–2.71)⁎	0.89	(0.61–1.30)	1.06	(0.78–1.56)
Two stressors	2.47	(1.59–3.83)⁎⁎⁎	0.99	(0.68–1.44)	1.18	(0.84–1.66)
Three stressors	2.45	(1.49–4.02)⁎⁎⁎	1.59	(1.06–2.39)⁎	1.31	(0.88–1.95)
Four or more stressors	3.80	(2.18–6.62)⁎⁎⁎	1.85	(1.13–3.03)⁎	2.46	(1.57–3.85)⁎⁎⁎
Model 2						
No stressors (ref.)	1		1		1	
One stressor	1.75	(1.10–2.78)⁎	0.95	(0.64–1.39)	1.13	(0.80–1.60)
Two stressors	2.46	(1.58–3.84)⁎⁎⁎	1.02	(0.69–1.49)	1.15	(0.82–1.63)
Three stressors	2.56	(1.55–4.23)⁎⁎⁎	1.65	(1.09–2.49)⁎	1.34	(0.90–2.01)
Four or more stressors	4.09	(2.32–7.21)⁎⁎⁎	1.85	(1.11–3.09)⁎	2.63	(1.66–4.18)⁎⁎⁎
Model 3						
No stressors (ref.)	1		1		1	
One stressor	1.77	(1.11–2.82)⁎	0.83	(0.56–1.24)	0.91	(0.63–1.32)
Two stressors	2.47	(1.58–3.87)⁎⁎⁎	0.84	(0.57–1.24)	0.91	(0.63–1.31)
Three stressors	2.64	(1.60–4.38)⁎⁎⁎	1.39	(0.91–2.13)	1.01	(0.66–1.54)
Four or more stressors	4.07	(2.31–7.19)⁎⁎⁎	1.31	(0.77–2.24)⁎	1.68	(1.03–2.74)⁎

*Stressors at 43 years*						
Model 1 (unadjusted)						
No stressors (ref.)			1		1	
One stressor			1.55	(0.99–2.44)	1.37	(0.98–1.90)
Two stressors			3.39	(2.19–5.24)⁎⁎⁎	1.60	(1.13–2.28)⁎⁎
Three stressors			5.93	(3.76–9.37)⁎⁎⁎	2.28	(1.55–3.36)⁎⁎⁎
Four or more stressors			7.78	(4.66–12.99)⁎⁎⁎	2.70	(1.70–4.28)⁎⁎⁎
Model 2						
No stressors (ref.)			1		1	
One stressor			1.47	(0.93–2.33)	1.35	(0.97–1.90)
Two stressors			3.36	(2.16–5.22)⁎⁎⁎	1.64	(1.15–2.35)⁎⁎
Three stressors			5.80	(3.64–9.25)⁎⁎⁎	2.29	(1.54–3.40)⁎⁎⁎
Four or more stressors			7.44	(4.41–12.57)⁎⁎⁎	2.58	(1.61–4.12)⁎⁎⁎
Model 3						
No stressors (ref.)			1		1	
One stressor			1.41	(0.89–2.26)	1.20	(0.84–1.71)
Two stressors			3.19	(2.04–4.99)⁎⁎⁎	1.19	(0.82–1.74)
Three stressors			5.24	(3.26–8.43)⁎⁎⁎	1.38	(0.90–2.10)
Four or more stressors			6.29	(3.69–10.73)⁎⁎⁎	1.28	(0.77–2.12)

Note: Model 1 unadjusted; Model 2 adjusts for gender, educational attainment, occupational social class, and relationship status; Model 3 further adjusts for past common mental disorder. ⁎*p* < 0.05; ⁎⁎*p* < 0.01; ⁎⁎⁎*p* < 0.001.

**Table 4:** Unadjusted and adjusted odds ratios (OR, 95% C.I.) for scoring above the cut off score on common mental disorder outcomes by subsequent life change and differentiating by type at 36 or 43 years (*N* = 2073).

			PSE at 36 years		PSF at 43 years		GHQ-28 at 53 years
		OR	(C.I.)	OR	(C.I.)	OR	(C.I.)
*Stressors at 36 years*							
Model 1 (unadjusted)							
Family and Economic	No stressors	0.75	(0.51–1.09)	1.01	(0.70–1.46)	0.87	(0.63–1.19)
	Stressors, no life change (ref.)	1		1		1	
	Stressors, with life change	2.05	(1.33–3.16)⁎⁎⁎	1.68	(1.07–2.63)⁎	1.11	(0.73–1.67)
Interpersonal Relationship	No stressors	0.72	(0.51–1.02)	0.95	(0.70–1.29)	0.87	(0.66–1.15)
	Stressors, no life change (ref.)	1		1		1	
	Stressors, with life change	1.64	(1.16–2.31)⁎⁎	1.48	(1.06–2.08)⁎	1.68	(1.24–2.28)⁎⁎⁎
Model 2							
Family and Economic	No stressors	0.69	(0.47–1.01)	0.98	(0.67–1.44)	0.80	(0.58–1.11)
	Stressors, no life change (ref.)	1		1		1	
	Stressors, with life change	1.97	(1.26–3.08)⁎⁎	1.57	(0.99–2.49)	1.07	(0.70–1.63)
Interpersonal Relationship	No stressors	0.71	(0.50–1.01)	0.91	(0.66–1.24)	0.88	(0.66–1.17)
	Stressors, no life change (ref.)	1		1		1	
	Stressors, with life change	1.60	(1.11–2.24)⁎	1.42	(1.00–2.01)⁎	1.65	(1.21–2.25)⁎⁎
Model 3							
Family and Economic	No stressors	0.70	(0.48–1.03)	1.06	(0.72–1.55)	0.90	(0.64–1.26)
	Stressors, no life change (ref.)	1		1		1	
	Stressors, with life change	1.98	(1.27–3.10)⁎⁎	1.29	(0.80–2.07)	0.87	(0.56–1.35)
Interpersonal relationship	No stressors	0.70	(0.49–0.99)⁎	0.97	(0.71–1.33)	0.99	(0.74–1.34)
	Stressors, no life change (ref.)	1		1		1	
	Stressors, with life change	1.58	(1.11–2.24)⁎	1.26	(0.88–1.81)	1.42	(1.02–1.97)⁎

*Stressors at 43 years*							
Model 1 (unadjusted)							
Family and economic	No stressors			0.42	(0.31–0.57)⁎⁎⁎	0.71	(0.54–0.93)⁎
	Stressors, no life change (ref.)			1		1	
	Stressors, with life change			1.51	(1.07–2.12)⁎⁎	1.08	(0.77–1.53)
Interpersonal relationship	No stressors			0.65	(0.47–0.89)⁎⁎	0.74	(0.57–0.96)⁎
	Stressors, no life change (ref.)			1		1	
	Stressors, with life change			2.12	(1.53–2.93)⁎⁎⁎	1.57	(1.15–2.14)⁎⁎
Model 2							
Family and economic	No stressors			0.40	(0.29–0.55)⁎⁎⁎	0.71	(0.54–0.93)⁎
	Stressors, no life change (ref.)			1		1	
	Stressors, with life change			1.43	(1.01–2.03)⁎⁎	1.07	(0.75–1.51)
Interpersonal relationship	No stressors			0.66	(0.48–0.91)⁎	0.72	(0.55–0.94)⁎
	Stressors, no life change (ref.)			1		1	
	Stressors, with life change			2.03	(1.46–2.84)⁎⁎⁎	1.45	(1.05–1.98)⁎
Model 3							
Family and economic	No stressors			0.42	(0.30–0.58)⁎⁎⁎	0.92	(0.69–1.23)
	Stressors, no life change (ref.)			1		1	
	Stressors, with life change			1.37	(0.96–1.96)	0.94	(0.65–1.36)
Interpersonal relationship	No stressors			0.67	(0.48–0.92)⁎	0.82	(0.61–1.08)
	Stressors, no life change (ref.)			1		1	
	Stressors, with life change			1.93	(1.37–2.71)⁎⁎⁎	1.23	(0.89–1.71)

Note: Model 1 unadjusted; Model 2 adjusts for gender, educational attainment, occupational social class, and relationship status; Model 3 further adjusts for past common mental disorder. ⁎*p* < 0.05; ⁎⁎*p* < 0.01; ⁎⁎⁎*p* < 0.001.

**Table 5:** Adjusted odds ratios (OR, 95% C.I.) for scoring above the cut off score on the GHQ–28 at 53 years by subsequent life change and differentiating by type at 36 and 43 years (*N* = 2073).

		Model 1		Model 2
	OR	(C.I.)	OR	(C.I.)
*Stressors at 36 years*				
Family and economic				
None	0.84	(0.60–1.16)	0.90	(0.64–1.27)
Stressors, no life change (ref.)	1		1	
Stressors, with life change	1.04	(0.68–1.59)	0.87	(0.56–1.35)
Interpersonal relationship				
None	0.94	(0.70–1.25)	1.02	(0.75–1.37)
Stressors, no life change (ref.)	1		1	
Stressors, with life change	1.59	(1.16–2.17)⁎⁎	1.41	(1.02–1.96)⁎

*Stressors at 43 years*				
Family and economic				
No stressors	0.72	(0.55–0.95)⁎	0.92	(0.69–1.23)
Stressors, no life change (ref.)	1		1	
Stressors, with life change	1.02	(0.72–1.45)	0.92	(0.63–1.32)
Interpersonal relationship				
No stressors	0.73	(0.56–0.96)⁎	0.82	(0.61–1.08)
Stressors, no life change (ref.)	1		1	
Stressors, with life change	1.41	(1.03–1.93)⁎	1.21	(0.87–1.69)

Note: Model 1 adjusts for gender, educational attainment, occupational social class, and relationship status; Model 2 further adjusts for past common mental disorder. ⁎*p* < 0.05; ⁎⁎*p* < 0.01; ⁎⁎⁎*p* < 0.001.
